# Collectanea Medica: Consisting of Anecdotes, Facts, Extracts, Illustrations, &c.

**Published:** 1824-11

**Authors:** 


### COLLECTANEA MEDICA:

CONSISTING OF

### ANECDOTES, FACTS, EXTRACTS, ILLUSTRATIONS, &c.

*Relating to the History or the Art of Medicine, and the Collateral Sciences.*

Floriferis, ut apes, in saltibus omnia libant,
Omnia nos, itidem, depascimur aurea dicta.

ART. I. — *Of Semi-decussation of the Optic Nerves.* By WILLIAM HYDE WOLLASTON, M.D. V.P.R.S.—(From the Philosophical Transactions for 1824, Part I.)

WHETHER we consider the astonishing subtlety of that medium which renders visible to us objects existing at the most immeasurable distances from us, or that delicately constituted organ which, by its general structure, collects the rays of light, and, by a nice adaptation of its parts, concentrates their force on the sentient fibres of the retina, expanded over its inner surface, we can feel no surprise that such great talents should have been devoted to investigate the curious properties of the one, or that the structure of the other should have been examined with so much assiduity.

The keenness of inquiry manifested by the cultivators of anatomy, in observing the most minute parts that have escaped the notice of their predecessors, shows that any addition to the common stock of our information on this subject will be gratifying to a certain portion of the members of this Society, and probably not uninteresting to the Society at large.

It is not my object, in the present paper, to examine either the first effect of the cornea in rendering the rays of light convergent, or the power of the crystalline lens in finally bringing them to a focus on the retina. It is not my intention to investigate whether the adaptation of the eye to different distances is effected by alteration of the form of the lens from its own muscular structure, or by alteration of its place, from the agency of other muscles. Nor do I mean to consider either the *involuntary* motions of the iris dependent on the quantity of light present, or that *voluntary* contraction of it by which we adapt the aperture of the pupil for distinct vision at different distances, limiting thereby, what in optics is termed the spherical aberration of the lens.

The subject of my inquiry relates solely to the course by which impressions from images perfectly formed are conveyed to the sensorium, and to that structure and distribution of the optic nerves on which the communication of these impressions depends.

Without pretending to detect, by manual dexterity as an anatomist, the very delicate conformation of the nerves of vision, I have been led, by the casual observation of a few instances of diseased vision, to draw some inferences respecting the texture of that part which has been called the decussation of the optic nerves, upon which I feel myself warranted to speak with some confidence.

It is well known that, in the human brain, these nerves, after passing forwards to a short distance from their origin in the thalami nervorum opticorum, unite together, and are, to appearance, completely incorporated; and that from this point of union proceed two nerves, one to the right, the other to the left eye.

The term decussation was applied to this united portion, under the supposition that, though the fibres do intermix, they still continue onward in their original direction, and that those from the right side cross over wholly to supply the left eye, while the right eye is supplied entirely from fibres arising from the left thalamus.

In this opinion, anatomists have felt themselves confirmed by the result of their examination of other animals, and especially that of several species of fish, in which it is distinctly seen that the nerves do actually cross each other as a pair of separate cords, lying in contact at their crossing, but without any intermixture of their fibres.

In these cases it is most indisputably true, that the eye upon the right side of the animal does receive its optic nerve from the left side of the brain, while that of the left eye comes from the right side; but it is not a just inference to suppose the same continuity preserved in other animals, where such complete separation of the entire nerve is not found.

On the contrary, I not only see reason, from a species of blindness which has happened to myself more than once, to conclude that a different distribution of nerves takes place in us, but I think my opinion supported by this evident difference of structure in fishes.

It is now more than twenty years since I was first affected with the peculiar state of vision to which I allude, in consequence of violent exercise I had taken for two or three hours before. I suddenly found that I could see but half the face of a man whom I met; and it was the same with respect to every object I looked at. In attempting to read the name *Johnson*, over a door, I saw only *son*; the commencement of the name being wholly obliterated to my view. In this instance the loss of sight was towards my left, and was the same whether I looked with the right eye or the left. This blindness was not so complete as to amount to absolute blackness, but was a shaded darkness without definite outline. The complaint was of short duration, and in about a quarter of an hour might be said to be wholly gone; having receded with a gradual motion from the centre of vision obliquely upwards toward the left.

Since this defect arose from over-fatigue, a cause common to many other nervous affections, I saw no reason to apprehend any return of it, and it passed away without need of remedy, without any further explanation, and without my drawing any useful inference from it.

It is now about fifteen months since a similar affection occurred again to myself, without my being able to assign any cause whatever, or to connect it with any previous or subsequent indisposition. The blindness was first observed, as before, in looking at the face of a person I met, whose left eye was to my sight obliterated. My blindness was in this instance the reverse of the former, being to my right (instead of the left) of the spot to which my eyes were directed; so that I have no reason to suppose it in any manner connected with the former affection.

The new punctum cæcum was situated alike in both eyes, and at an angle of about three degrees from the centre; for, when any object was viewed at the distance of about five yards, the point not seen was about ten inches distant from the point actually looked at.

On this occasion the affection, after having lasted with little alteration for about twenty minutes, was removed suddenly and entirely, by the excitement of agreeable news respecting the safe arrival of a friend from a very hazardous enterprise.

In reflecting upon this subject, a certain arrangement of the optic nerves has suggested itself to me, which appears to afford a very probable interpretation of a set of facts, which are not consistent with the generally-received hypothesis of the decussation of the optic nerves.

Since the corresponding points of the two eyes sympathise in disease, their sympathy is evidently from structure, not from mere habit of feeling together, as might be inferred, if reference were had to the reception of ordinary impressions alone. Any two corresponding points must be supplied with a pair of filaments from the same nerve, and the seat of a disease in which similar parts of both eyes are affected must be considered as situated at a distance from the eyes, at some place in the course of the nerves where these filaments are still united, and probably in one or the other thalamus nervorum opticorum.

It is plain that the cord, which comes finally to either eye, under the name of optic nerve, must be regarded as consisting of two portions,—one half from the right thalamus, and the other from the left thalamus nervorum opticorum.

According to this supposition, decussation will take place only between the adjacent halves of the two nerves. That portion of nerve which proceeds from the right thalamus to the right side of the right eye, passes to its destination without interference; and, in a similar manner, the left thalamus will supply the left side of the left eye with one part of its fibres, while the remaining halves of both nerves, in passing over to the eyes of the opposite side, must intersect each other, either with or without intermixture of their fibres.

Now, if we consider rightly the facts discovered by comparative anatomy in fishes, we shall find that the crossing of the entire nerves in them to the opposite eyes, is in perfect conformity to this view of the arrangement of the human optic nerves. The relative position of the eyes to each other in the sturgeon, is so exactly back to back, on opposite sides of the head, that they can hardly see the same object; they can have no points which generally receive the same impressions, as in us; there are no corresponding points of vision requiring to be supplied with fibres from the same nerve. The eye which sees to the left has its retina solely upon its right side, and this is supplied with an optic nerve arising wholly from the right thalamus; while the left thalamus sends its fibres entirely to the left side of the right eye, for the perception of objects situated on the right. In this animal, an injury to the left thalamus might be expected to occasion entire blindness of the right eye alone, and want of perception of objects placed on that side. In ourselves, a similar injury to the left thalamus would occasion blindness (as before) to all objects situated to our right, owing to insensibility of the left half of the retina of both eyes.

A disorder that has occurred within my own knowledge in the case of a friend, seems fully to confirm this reasoning, as far as a single instance can be depended upon. After he had suffered severe pain in his head for some days, about the left temple, and toward the back of the left eye, his vision became considerably impaired, attended with other symptoms indicating a slight compression on the brain.

It was not till after the lapse of three or four weeks that I saw him, and found that, in addition to other affections which need not here be enumerated, he laboured under a defect of sight similar to those which had happened to myself, but more extensive, and it has unfortunately been far more permanent. In this case the blindness was at that time, and still is, entire, with reference to all objects situated to the right of his centre of view. Fortunately, the field of his vision is sufficient for writing perfectly. He sees what he writes, and the pen with which he writes, but not the hand that moves the pen. This affection is, as far as can be observed, the same in both eyes, and consists in an insensibility of the retina on the left side of each eye. It seems most probable that some effusion took place at the time of the original pain on that side of the head, and has left a permanent compression on the left thalamus. This partial blindness has now lasted so long without sensible amendment, as to make it very doubtful when my friend may recover the complete perception of objects on that side of him.

In reviewing the several phenomena that I have described, we find partial blindness occurring at the same time in both eyes. This sympathy from disease is readily explained, on the supposition that the parts which sympathize receive their nerves from the same source, while the opposite halves of the eyes, which are not at the same time similarly affected, are supplied from an opposite source; and the inference is immediate, that, in common vision also, the sympathy of corresponding points, which receive similar impressions from the same object, is dependent on the arrangement of nerves thus detected by disease.

We find moreover in the sturgeon, (and it is the same in some other fishes,) whose eyes can scarcely see the same object at once, and have no corresponding points which ordinarily sympathize, that the two eyes do not receive any nervous fibres from the same source; but one eye receives its nerve wholly from one side, and the other from the other side of the brain.

From the structure of these fish, we learn distinctly that the perception of objects toward one side is dependent on nerves derived from the opposite side of the brain; and, in the last case of diseased vision above related, we find apparent injury to one side of the brain followed by blindness toward the opposite side of the point to which both eyes are directed.

A series of evidence in such apparent harmony throughout, seems clearly to establish that distribution of nerves I have endeavoured to describe, which may be called the semi-decussation of the optic nerves.

*On single Vision with two Eyes.*

So long as our consideration of the functions of a pair of eyes is confined to the performance of healthy eyes in common vision, when we remark that only one impression is made upon the mind, though two images are formed at the same moment on corresponding parts of our two eyes, we may rest satisfied in ascribing the apparent unity of the impression to habitual sympathy of the parts, without endeavouring to trace further the origin of that sympathy, or the reason why, in infancy, the eyes ever assume one certain direction of correspondence, in preference to squinting.

But, when we regard sympathy as arising from structure, and dependent on connexion of nervous fibres, we therein see a distinct origin of that habit, and have presented to us a manifest cause why infants first begin to give the corresponding direction to their eyes, and we clearly gain a step in the solution, if not a full explanation, of the long agitated question of single vision with two eyes.

It may, perhaps, to some persons appear surprising that so many as three instances of a disorder, which they presume to be rare, should have been witnessed by one individual; but I apprehend, on the contrary, this half-blindness to be far more common than is generally supposed; and I might, with as much reason, express surprise at its having so far escaped notice,* were I not aware how many facts commonly remain disregarded, merely for want of explanation. It is evident that I once, and for a long time, overlooked the inference that is to be drawn from this affection; and, if the disorder had not happened to me a second time, I might never have reconsidered its cause.

Even since the preceding pages were written, I have met with two more cases of this disease. One of my friends has been habitually subject to it for sixteen or seventeen years, whenever his stomach is in any considerable degree deranged. In him the blindness has been invariably to his right of the centre of vision, and, from want of due consideration, had been considered as temporary insensibility of the right eye; but he is now satisfied that this really is not the case, but that both eyes have been similarly affected with half-blindness. This symptom of his indigestion usually lasts about a quarter of an hour or twenty minutes, and then subsides, without leaving any permanent imperfection of sight.

I have not seen the subject of the fifth case; but I am informed that he has had many returns of this affection, generally attended with head-ache, and always lasting about twenty minutes, with very little variation.

---

* Richter, in the third volume of his "Elements of Surgery," has a chapter on Half-blindness, and part of it relates to what he terms Amaurosis dimidiata. From one instance there given, he seems to have seen some cases similar to those I have described; but he has not noticed the corresponding affection of the two eyes, or considered the sympathy between them.—*Anfangs-gründe der Wundartzeneykunst*, vol. iii. chap. 16, p. 478.

**Art. II.**—*Effects of Lightning upon the Human Body, exemplified by a remarkable Accident.* By Dr. Tilesius, of Mülhausen.—(From the Edinburgh Philosophical Journal.)

In the ninth volume of Schweigger's Journal, there is an account of an accident, caused by lightning, which may throw some light upon the mode of action of this formidable meteor. As two carts were proceeding in a hollow way, bordered on either side by a wood, they were successively struck by a thunder-bolt. In the first cart were seated the two brothers Teele, the one aged thirty-three years, the other twenty-nine: in the second, Mr. Teele the nephew, a young man of twenty, and Mr. Decker. The lightning struck successively the horse of the first cart, the two brothers, Mr. Decker, and his companion; the latter of whom did not survive the accident. The horse was killed upon the spot: the skin of the belly was torn in all the lower part, the mouth left open, and the teeth blackened. It struck the younger Teele, passing through his umbrella, which was thrown to a distance of twenty-four paces from the cart: the cart itself was perforated with a hole of half a foot in diameter. The body, on being carried to the nearest village, was put into a tepid bath, and rubbed; blood flowed from the nose, the mouth, and ears, but no sign of life appeared. The mouth and nose were blackened; the skin and muscles of the arms and hands, which were both employed in holding the shaft of the umbrella, were furrowed to the bone; the sleeves of the coat and shirt were torn; but the lesions of the skin were not of the nature of tumors or scars, such as are produced by the application of red-hot iron: the skin looked as if it had been raised by a very quick rubbing. In the same manner, the clothes bore no marks of burning, but seemed as if they had been torn by the rapid passage of a sharp point. Mr. Decker, who was in the same cart, received, at the same instant, so violent a blow in the lower belly, that he was precipitated from the cart, and remained senseless for half an hour. When he was undressed, the place in which he had felt the shock was of a bright red colour, but without any open wound. He was, by this time, in a condition for continuing his journey.

The two brothers Teele had suffered considerable damage from the lightning; they, however, quickly recovered, as will presently be seen. But it will be interesting, in the first place, to follow the progress of the electric fluid over the different parts of their bodies, and to observe the nature of the wounds which resulted. They were sitting by the side of one another when struck. The lightning first hit the head of the elder; it tore to pieces the velvet cap which he had on, grazed the temporal bone an inch above the left ear, and then behind the ear; after which, slightly raising the skin, it descended upon the neck, traversing the back part obliquely, and ascended toward the right ear; here it scratched the inner part of the ear, near the tragus and antihelix; it then fell upon the right shoulder, passed beneath the chin, over the right breast and arm; and, returning to the back, descended along the vertebral column to the sacrum. In this latter part of the course of the lightning, the skin was not cut, but only a little raised, and very red. Impressions of the same nature were seen across the arms; and attested, as well as the rupture of the clothes, the zig-zag progress of the lightning, which had passed alternately from the right side of the younger brother to the left side of the elder. It fixed upon the latter, on meeting with some pieces of metal that were in his waistcoat pocket: here it raised the skin upon a space about the size of the hand. After this it descended upon the left part of the region of the pubis, and traversed the inner surface of the thigh, the ham, and calf of the leg. A piece of steel, which the younger of the brothers carried in his fob, led the lightning to the region of the groin, where a space of the size of the piece was deprived of the skin, and affected with a deep wound. The breadth of the mark left by the lightning upon the different parts of the body was in general two inches; the wounds were more extended and deeper at the intersections of this mark; several of them were very painful, and suppurated abundantly. The skin had been rolled, in close folds, to the right and left, by the rapid passage of the lightning. The wounds did not bleed; and all that had to be done was to provide for the renovation of the skin destroyed. In a word, there was no indication of any lesion of organs by fire or heat; but the effect produced might be compared to that which takes place when a ball grazes the surface of a limb.

Dr. Tilesius having assisted at the two first dressings, had all the leisure necessary for carefully examining the form and nature of the hurts: he even took a sketch of them, which accompanies his Memoir.

The brothers Teele, after having perfectly recovered themselves, were affected with violent nausea, and vomited repeatedly, when some cups of tea were given them to drink: they threw up a little blood at first, as had happened to the one who had been killed. Notwithstanding the great extent of their wounds, and their being besides of a robust habit, they had no fever. The elder was perfectly deaf on the day of the accident; but on the following day he recovered his hearing to a certain degree. No trace of paralysis made its appearance in the limbs struck by the lightning. The wounds were cicatrized in the space of a few weeks.

Dr. Tilesius having seen Dr. Bauer, the physician of the brothers Teele, a year after the accident, (which took place in May 1821,) received from him the following information:—The elder has remained somewhat dull of hearing, more or less so according to the season: he experiences a marked disposition to sleep, and would often remain twenty-four hours together asleep, were he not wakened. The younger has latterly had an inflammatory fever. He is subject to a periodical weakness, or state of relaxation, which was before unknown to him. In general, it has had a much greater influence upon the nervous system of the two brothers than might have been presumed from the vigour of their constitution. The cicatrices of the wounds now present, in several places, the appearance of the turns of a screw.

ART. III.—*Account of William Dempster, who swallowed a Table-knife nine inches long; with a Notice of a similar Case in a Prussian Knife-eater.* By THOMAS BARNES, M.D. Member of the Royal College of Surgeons, London; Member of the Wernerian Natural History Society; and Physician to the Fever Hospital and Dispensary, Carlisle.—(From the Edinburgh Philosoph. Journal.)

SEVERAL cases of knife-eaters are on record. One of the most remarkable is that of John Cummings, who lived ten years after having swallowed a number of clasp-knives: his case is related by Mr. Marcet, and an account of it is published in the seventh volume of the Edinburgh Philosophical Journal. The following case lately occurred in Carlisle, and excited considerable interest and sympathy, not only among the inhabitants of that city, but also very generally throughout the country. The case was particularly interesting to the medical profession, both as to the physiological fact that the functions of life were not interrupted, and even suffered little disturbance, by the presence of so large an extraneous substance as a table-knife in the stomach; and as to the medical and chirurgical treatment the profession could supply in so singular a case.

William Dempster, a juggler, twenty-eight years of age, of a high complexion and sanguine temperament, came to Carlisle in November last, with the intention of exhibiting some tricks by slight-of-hand; and, on the evening of the 17th of the same month, when in a small inn in Botchergate, with a number of people about him, whom he was amusing by pretending to swallow a table-knife; and, in the act of putting the knife into his throat, he thought some person near him was about to touch his elbow, which agitated and confused him so much, that the knife slipped from his fingers, and passed down the gullet into the stomach. Immediately after the accident, he became dreadfully alarmed, was in great mental agony, and apprehended instantaneous death. The knife, when given to him, measured nine inches in length, and had a bone handle, which went first down into the stomach: the blade, which was not very sharp, was one inch in breadth. Medical assistance was soon procured, and several attempts were made to extract the knife: first with the fingers alone, then with a pair of short curved forceps, and afterwards by a pair of very long forceps, made for the occasion, but without success. The knife, indeed, could not be reached by any of these means; and nothing resembling it could be felt externally on the region of the stomach. His mind continued much depressed, though he had very little pain or uneasiness. He was encouraged by the medical attendants, and directed to be removed as quietly as possible to his lodgings, and to take nothing that night except a little cold water. He had some sleep, and next morning said he felt occasionally pain in his stomach. Twelve ounces of blood were taken from his arm, and an enema was ordered him. He afterwards complained of pain in the left shoulder, shooting across the chest to the stomach; and the blood-letting was repeated. A hard substance, which was believed to be the handle of the knife, could now be felt very distinctly, by pressing the fingers very gently on the umbilicus; slight pressure gave him considerable pain. Although his sufferings was much less than could have been expected, his health became gradually impaired, and his strength reduced. He was able to walk about a little in the day, and could sleep in the night on his back, but could not lie on either side. He took some diluted sulphuric acid for two or three weeks, which was discontinued, as he thought it increased the pain in his stomach. His bowels were kept open by castor-oil and injections; the alvine evacuations were of a dark ferruginous colour, which probably arose from the decomposition of the knife. The pulse was very little affected, being generally between seventy and eighty in a minute. His diet consisted of soup, gruel, and tea, taken in small quantities. When the stomach was empty of food, the handle of the knife could be distinctly felt, extending from above downwards, by placing the hand very lightly on the abdomen, a little above the umbilicus; but a single cup of tea, or a little food of any kind, distended the stomach so much, that it entirely disappeared. He was frequently squeamish and sick at his stomach, and sometimes felt a severe twisting pain in that organ.

The case being a remarkable one, and of very rare occurrence, the patient was visited by a great number of medical men. All the professional men in Carlisle were consulted respecting him; and, that nothing might be omitted that could benefit this unfortunate man, his case was stated to Sir Astley Cooper of London, Mr. George Bell of Edinburgh, and a few others. As the great length of the knife would prevent the possibility of its passing the pylorus, or making the turns of the intestines, and it seemed improbable that the patient would live sufficiently long for it to be dissolved in the stomach, various means were suggested to extract it; for, although Dempster had survived the first shock of swallowing the knife, and there was no risk of speedy destruction of life, the action of the gastric juice, or of any medicine that could be given, it was supposed would be so slow, particularly upon the blade of the knife, that it was deemed advisable to extract it, if possible. Besides the means already mentioned, the following (though not had recourse to) was deserving of notice:—An eminent and excellent surgeon recommended that Dempster should be accustomed to receive, two or three times a-day, a large smooth elastic gum bougie into the stomach, and gradually allow it to remain one, two, three, or ten minutes there; that tubes of elastic gum, twenty inches long, should be prepared, of various sizes, from one-quarter of an inch in diameter, and open at each end; that the extremity to be introduced into the stomach should be filled up by means of an ivory ball attached to a wire or piece of whalebone, so that the lining membrane of the œsophagus would not be injured during its passage. The piece of ivory to be removed, and instead of it a pair of forceps, resembling those used by Sir Astley Cooper for removing stones from the bladder, or a pair of forceps which expand of themselves when pushed forward and not restrained by the tube, to be introduced. The same surgeon observed, that many contrivances might be devised for laying hold of, or entangling, the knife; but, as it might be seized at an improper place, no instrument should be used but which could be immediately disengaged from the knife, if necessary.

The above plan was recommended on the principle, that when a probang, or any foreign instrument, is forcibly introduced into the stomach through the œsophagus, violent exertions and spasms of the muscles concerned in deglutition take place; but, on every successive repetition, these spasms become less and less, until they abate almost entirely. This is exemplified in those who require to be fed by a tube, introduced into the stomach, and a syringe.

Another plan was to make the knife force its own way through the parietes of the stomach and abdomen, and so assist it ultimately by a chirurgical operation. This was to be accomplished by the patient's lying entirely on one side or on his face, when his stomach was empty, so that inflammation and suppuration might be excited; and, after adhesion had taken place, might be aided by the scalpel. We know that a mulberry calculus has repeatedly forced its way through the bladder and rectum, through the bladder and perineum, through the bladder above the pubes, by the patient having been confined for years to bed, and lying in the relative postures favourable for such operations of nature; also, that many gall-stones have forced their way through the abdominal parietes.

The only other plan of treatment that I shall mention, is that which was proposed by the surgeons of the Carlisle Dispensary, and was also recommended and sanctioned by one of the first surgeons in Europe: it was, that an incision should be made into the patient's stomach, and the knife extracted. The last report of the Carlisle Dispensary contains the following observations concerning Dempster:—"The surgeons of the Dispensary were unanimously agreed as to the best mode of treating this extraordinary case: they were of opinion that nothing but an operation could save the patient's life, but he could not be persuaded to submit to it."

He remained in Carlisle until the 28th of December, when he left it, with the intention of proceeding to his friends at Hammersmith, in the neighbourhood of London. It is proper to remark, that his journey was neither recommended nor sanctioned by the medical officers of the Dispensary; it was contrary to their advice: they apprehended dangerous and fatal consequences from it, and anxiously wished him to continue in Carlisle. It appears from the daily prints, that what they apprehended has in reality happened. This unfortunate man was prevented from pursuing his journey further than Middlewick in Cheshire, where he died on the 16th of January; inflammation and gangrene of the stomach having been produced by the irritation of the knife, and the jolting of the conveyance in his journey.

The celebrated surgeon above alluded to, who recommended an operation, stated, that he was decidedly of opinion that an incision should be made into the person's stomach, if the handle of the knife could be felt as the director to the surgeon. The incision should be in the direction of the linea semilunaris. The stomach should be previously quite empty of food and of liquids. The patient was to be supported, for ten days after the operation, by glysters of broth and jelly; after ten days, jelly might be given.

As Dempster died at a considerable distance from Carlisle, and no authentic account of the dissection has been published, I am not aware of what change the knife had undergone by being retained in the stomach, nor of the precise appearances the abdominal viscera exhibited after death. The present communication, it is hoped, will induce the surgeons who examined the body, to give an account of their dissection to the public.

A case very similar to the above occurred in Prussia in 1635, of which a very interesting account was written in Latin, by Dr. Daniel Beckher, of Dantzic, and published at Leyden in 1636. The case is well authenticated. Beckher's account of it was submitted to the Faculty of Leyden, who affixed their names to their criticism of his work. It received from them unqualified praise. They passed many high encomiums on the author; considered the case singular, the cure miraculous, and the history of it faithfully and accurately detailed. The style of the work is elegant and classical; the case is described with much minuteness, simplicity, and clearness, and is accompanied with many excellent and valuable observations. The book is divided into four sections. The first treats of the swallowing of the knife; the second, the consultation of the Faculty; the third, the incision of the abdomen and stomach, and the extraction of the knife; and the fourth, the healing of the wound. The following is an abstract of the case:—On the morning of the 29th of May, 1635, Andrew Grunbeide, a young peasant, feeling sick at stomach from having committed some irregularity in his mode of living, endeavoured to excite vomiting by irritating the fauces with the handle of a knife; but the desired effect not being imdiately produced, he thrust it further down, in consequence of which it escaped his hold, and gradually descended into the stomach. The knife-eater was terribly frightened at the time, and continued afterwards much depressed, yet was able to follow his accustomed employment without much inconvenience. The wretched condition of this afflicted peasant excited much pity, and many physicians and surgeons of great learning and celebrity were consulted respecting him. At a meeting of the Faculty, held on the 25th of June, it was decided that the abdomen should be opened, an incision made into the stomach, and the knife extracted. Previous to the operation, the patient was to make use of a balsamic oil, called Spanish Balsam, which they supposed would alleviate the pains of the stomach, and facilitate the healing of the wound. The 9th of July was fixed for the operation; and it was performed in the presence of the dean of Faculty of Medicine, the physicians and members of College, the students of Medicine, and an experienced surgeon and lithotomist of the name of Shoval. A straight incision was made in the left hypochondrium, two fingers breadth, under the false ribs; first through the skin and cellular membrane, then through the muscles and peritoneum. The stomach subsided and slipped from the fingers, which prevented it from being immediately seized; but it was at length caught hold of with a curved needle, and drawn out of the wound. A small incision was then made into it upon the knife, which was then easily extracted. The stomach immediately collapsed. After the external wound had been properly cleaned, it was united with five sutures, and tepid balsam poured into the interstices. Tents impregnated with the same balsam, and a cataplasm composed of bolar earth, the white of egg, and alum, were then applied. In the evening, the cataplasm was removed, a styptic plaster applied, and he took a decoction of betony, tormentil, and fever-few, with a powder, consisting of nutmeg and crab's-eyes. The report next morning was, that he had had a quiet night; pulse a little accelerated; had passed some bloody urine, which deposited a sediment of grumous blood; the wound looked well; he complained of no pain. Two sutures were removed, and the balsam and plaster again applied. He was allowed chicken-broth, boiled with some bitter and astringent herbs. The wound was dressed again in the evening; and, as he had not had an alvine evacuation, a suppository was directed to be used. On the 11th, two more sutures were removed; pulse less frequent; urine still bloody; complained of pain and tension in the left hypochondrium. Two enemas were administered, and he passed a copious black dejection. The wound was regularly dressed twice a-day; he was allowed a very strict diet, and his drink was moderately warm; bowels were kept open by injections; urine continued tinged with blood until the 13th. On the 15th of July, and the seventh after the operation, he was pronounced out of danger. On the 16th, he took a little infusion of rhubarb, with syrup, as an aperient. The same treatment and dressing were continued until the 23d of July, which was the fourteenth day after the operation, when the wound had healed, and nothing occurred afterwards worthy of notice. He was restored to the best of health, gradually returned to his ordinary diet and employment, and never afterwards complained of pains in his stomach.

In the fifth volume of Jones's edition of the Philosophical Transactions, Dr. W. Oliver informs us, that in 1685 he was at Koningsberg in Prussia, where he saw the knife that was swallowed by the Prussian boor. It was kept in a velvet bag in the King of Prussia's library. According to the engravings given of it, he says it measured six inches and a half long, English measure. Beckher says, the knife "decem pollicum latitudinem adæquabat." Dr. Oliver met with a Mr. Taylor, a Scotch merchant at Koningsberg, who told him that Andrew Grunbeide was his particular friend and acquaintance; that he saw his wound several times when his surgeons dressed him, and was godfather to one or two of his children afterwards.

It is much to be regretted that William Dempster could neither be prevailed upon to submit to an operation, nor to remain in Carlisle. As an operation succeeded near two centuries ago, when surgery was in a very imperfect state, it is highly probable that, under the present improved state of surgery, a similar operation would have been attended with success. The many valuable improvements that have been introduced into surgery, both in the operative part and in the subsequent mode of treatment, must give the moderns a decided advantage over the ancients in the success of their operations. Had he remained in Carlisle, even although no operation had been performed, it is very probable his life would have been spared much longer than it actually was. He became weak and emaciated; but, as has been before stated, was able to walk about the town, and the stomach had, in some degree, become accustomed to the presence of the knife. The handle, and perhaps the blade also, would be dissolving, so that the bulk would be diminished; and, if the knife had not been altogether removed in this way, it would have produced less irritation, and he might have lived a considerable time. There is even some probability that the knife might, in the course of time, have made its way through the stomach and parietes of the abdomen, by inflammation, abscess, and ulceration; as extraneous bodies have frequently been brought from various internal parts to the external surface by these processes, or by what some surgeons have termed *progressive absorption.*

---

